# Biological Activity and Chemical Composition of Propolis from Various Regions of Poland

**DOI:** 10.3390/molecules28010141

**Published:** 2022-12-24

**Authors:** Magdalena Woźniak, Anna Sip, Lucyna Mrówczyńska, Justyna Broniarczyk, Agnieszka Waśkiewicz, Izabela Ratajczak

**Affiliations:** 1 Department of Chemistry, Faculty of Forestry and Wood Technology, Poznan University of Life Sciences, Wojska Polskiego 75, 60625 Poznań, Poland; 2 Department of Biotechnology and Food Microbiology, Faculty of Food Science and Nutrition, Poznan University of Life Sciences, Wojska Polskiego 48, 60627 Poznań, Poland; 3 Department of Cell Biology, Faculty of Biology, Adam Mickiewicz University, Uniwersytetu Poznańskiego 6, 61614 Poznań, Poland; 4 Department of Molecular Virology, Faculty of Biology, Adam Mickiewicz University, Uniwersytetu Poznańskiego 6, 61614 Poznań, Poland

**Keywords:** antimicrobial activity, antioxidant properties, antiviral activity, cytoprotective activity, phenolic compounds, propolis

## Abstract

Propolis is one of the bee products, with multiple biological properties used in numerous applications. The research objective was to determine the chemical composition and biological properties (antibacterial, antifungal, antiviral, antioxidant, and cytoprotective activity) of propolis extracts collected from various regions of Poland. The results indicated that the total content of phenols (116.16–219.41 mg GAE/g EEP) and flavonoids (29.63–106.07 mg QE/g EEP) in propolis extracts depended on their geographic origin. The high content of epicatechin, catechin, pinobanksin, myricetin, and acids: vanillic and syringic in propolis samples was confirmed by chromatographic analysis. Moreover, the presence of caffeic acid phenethyl ester was confirmed in all samples. The origin of propolis also influenced the biological properties of its extracts. The propolis extracts were characterized by moderate DPPH free radical scavenging activity (29.22–35.14%), and relatively low ferrous iron chelating activity (9.33–32.32%). The results indicated also that the propolis extracts showed high activity in the protection of human red blood cells against free radicals generated from 2,2’-azobis(2-methylpropionamidine) dihydrochloride (AAPH). The extracts exhibited diversified activity against the tested pathogenic bacteria and limited activity against fungal strains. The research of selected propolis extracts showed that only 2 of 5 examined samples showed moderate activity against HPV (human papillomaviruses) and the activity depended on its geographical distribution.

## 1. Introduction

Propolis is a natural resinous material collected by honey bees from buds and exudates of various plant species. In temperate climate zone, bees collect propolis resin, mainly from vegetive poplar buds, chiefly *Populus nigra*. However, other *Populus* genera, including *P. tremula* (aspen), could also be a precursor for propolis production [1,2,3]. Polish bee glue can also be produced by bees from the leaf buds of *Betula* spp. and *Alnus* spp. [4]. Propolis has been used since ancient times as a remedy in folk medicine [5]. Currently, the use of propolis is increasing and it is applied in many industries, including the cosmetology, pharmacy, medicine, and food industry.

Propolis consists of numerous chemical compounds such as polyphenols, terpenoids, amino acids, sugars, steroids, minerals, and vitamins [6,7,8,9,10,11]. Polyphenols, such as flavonoids, phenolic acids and their esters, phenolic alcohols, aldehydes, and ketones are important groups of propolis constituents, due to their wide biological activity [12,13,14]. Flavonoids, such as galangin, apigenin, pinocembrin, pinostrobin, kaempferol, chrysin, and quercetin, and also phenolic acids, including caffeic, ferulic, cinnamic, coumaric, and hydroxybenzoic were determined in propolis samples from various geographic origin [15,16,17,18]. The frequently identified compound in propolis from different counties, such as Italy, Spain, Poland, or India is caffeic acid phenethyl ester (CAPE), which possesses wide pharmacological effects, including activity against various types of cancer [18,19,20,21,22,23]. Literature data indicated that propolis collected from various parts of Poland is a rich source of phenols, such as chrysin, galangin, hesperidin, naringenin, quercetin, and aromatic acids: caffeic, ferulic, cinnamic, and genistic [1,3,4,16,24,25,26,27,28,29]. 

The many biological properties of propolis, including antibacterial, antifungal, antioxidant, anti-inflammatory, and anticancer have been described in the literature [30,31,32,33,34]. Moreover, the activity of propolis against various strains of viruses, such as herpes simplex virus (HSV-1), anti-influenza virus, anti-HIV-1, and anti-SARS-CoV-2 have been reported in the literature [35,36,37,38]. The research indicated also that propolis from various regions of Poland was characterized by antifungal, antibacterial, antioxidant, chemopreventive, and antiproliferative activities [1,4,24,27,28,29,39,40,41,42,43]. The chemical composition (total content of phenols and flavonoids, as well as phenolic profile) and thus biological activities of propolis extracts depend on many factors, such as the geographical origin of propolis sample, season of its collecting, bee species, and solvent used to extraction process or method of propolis harvesting [6,22,44,45,46,47,48,49]. 

The aim of the study was to determine the chemical composition and biological activity of ethanolic extracts of Polish propolis collected from various parts of the country. The chemical composition of propolis extracts was determined on the basis of the total content of phenols and flavonoids, as well as the concentration of individual phenolic compounds. Among the biological activity of propolis extracts, the antioxidant, antifungal, antibacterial, and cytoprotective effects against oxidative hemolysis of human erythrocytes in vitro were analyzed. Moreover, the activity of propolis extracts against HPV-16 (human papillomaviruses) using a pseudovirion system (PsVs) was determined. To the best of the authors’ knowledge, the activity of Polish propolis against the HPV-16 virus, as well as the influence of so many locations of obtaining Polish propolis on the chemical composition and biological activity of its extracts, have not been studied previously.

## 2. Results and Discussion

Propolis samples collected from apiaries located in various parts of Poland were used to study the chemical composition and biological activity of their ethanolic extracts. The locations of apiaries from which propolis was collected are presented in Section 3—Materials and Methods.

### 2.1. The Concentration of Phenolic Compounds

In propolis extracts, total phenolic content, total flavonoid content, and the concentration of individual phenolic compounds, including phenolic acids, flavonoids, and caffeic acid phenethyl ester were analyzed. 

#### 2.1.1. Total Phenolic Content (TPC) and Total Flavonoid Content (TFC)

**Figure 1:**
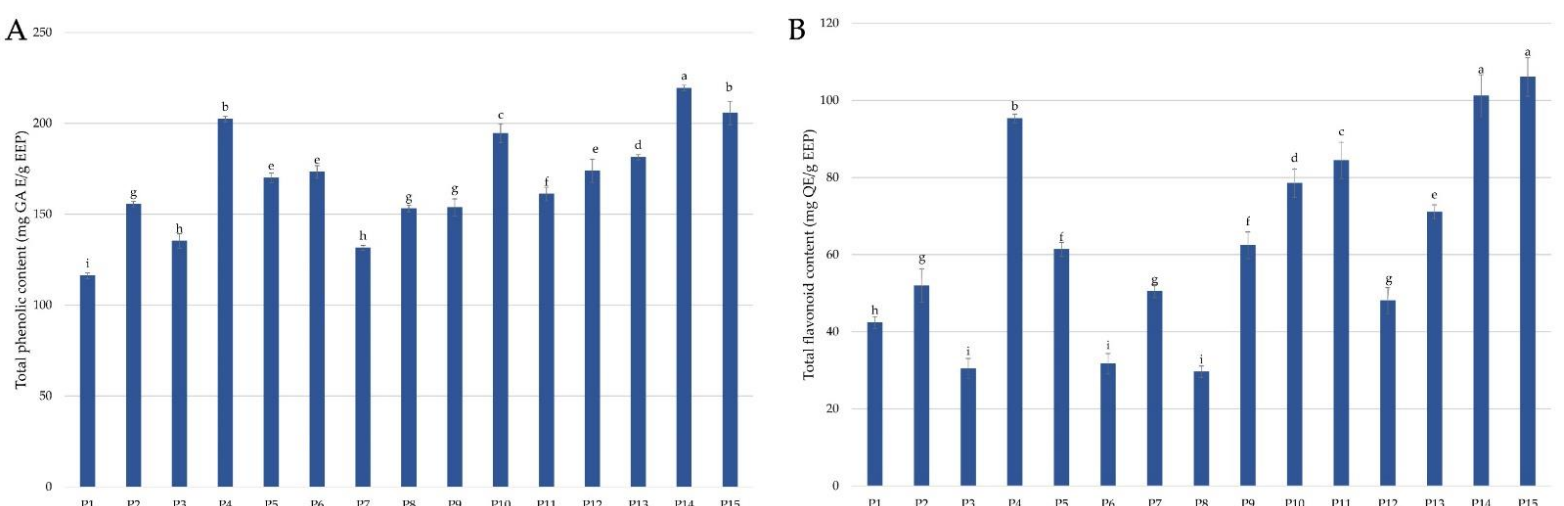
Total phenolic content (**A**) and total flavonoid content (**B**) in propolis extracts. (Values denoted with identical letters do not differ significantly).

The examined ethanolic propolis extracts (EEP) were characterized by different total phenolic content, depending on the place of propolis collection. The total phenolic contents in propolis extracts are presented in Figure 1A. 

The highest total phenolic content (219.41 mg gallic acid equivalent (GAE)/g EEP) was determined in the propolis extract collected in Greater Poland (P14). The propolis extracts from the West Pomeranian (P15) and from Łódzkie Province (P4) were also characterized by high total phenolic content, which was 205.64 and 202.32 mg GAE/g EEP, respectively. In turn, the lowest total phenolic content (116.16 mg GAE/g EEP) was detected in the propolis extract from Lower Silesia (P1). Low TPC values were also determined for extracts of propolis P3 collected from Lublin Province (135.26 mg GAE/g EEP) and propolis P7 harvested from an apiary located in Masovia (131.43 mg GAE/g EEP). 

The total phenolic contents in propolis extracts from Poland determined by Socha et al. [24] were in a range from 150.05 to 190.79 mg GAE/g of raw propolis and differed depending on the part of the country where propolis samples were collected. The highest TPC was determined in propolis from Kuyavian-Pomerania Province, while the lowest in propolis collected from Subcarpathian Province [24]. In turn, the total phenolic content in ethanolic extracts of propolis collected in three regions of Poland was assessed by Wezgowiec et al. [45]. TPC in propolis collected from Podlaskie Province was 220.05 mg GAE/g, in the sample harvested from Masovia—259.63 mg GAE/g, while TPC for propolis from West Pomerania Province was 275.79 mg GAE/g [45]. TPC in ethanolic extract of Polish propolis from West Pomerania was 150.80 mg GAE/g [40]. The extract of propolis from Germany was characterized by TPC equal to 46.45 mg caffeic acid equivalent (CAE)/g, propolis collected in the Czech Republic—129.83 mg CAE/g and Irish propolis—52.81 mg CAE/g [46]. Wang et al. [47] examined total phenolic content in 20 propolis samples from various regions of South Korea and found that samples contained TPC in a range from 48.5 to 238.9 mg GAE/g EEP. In turn, TPC of Australian propolis extract was 142.4 mg GAE/g EEP, Brazilian propolis—126.8 mg GAE/g EEP, and propolis from China was characterized by TPC equal to 132.1 mg GAE/g EEP [47]. Propolis samples from Morocco were characterized by TPC in a range from 1125 to 148 mg GAE/g, while propolis from Palestine in a range from 75 to 136 mg GAE/g [48]. TPC of aqueous extracts of propolis collected from different regions of Portugal ranged from 0.93 to 2.81 mg pinocembrin equivalent (PE)/mL, while methanolic extracts of propolis samples were characterized by TPC in a range from 3.39 to 8.76 mg PE/mL [49]. 

The total flavonoid contents in extracts of propolis collected from various regions of Poland are presented in Figure 1B. Values of total flavonoid content in the extracts of propolis collected from various parts of Poland ranged from 29.63 to 106.07 mg quercetin equivalent (QE)/g EEP. The highest total flavonoids content was detected for propolis P15 from West Pomerania (106.07 mg QE/g EEP) and propolis P14 from Greater Poland (101.22 mg QE/g EEP). The lowest TFC was determined for propolis P3 from Lublin Province (30.41 mg QE/g EEP), propolis P6 from Masovia (34.70 mg QE/g EEP) and propolis P8 collected from Subcarpathia Province (29.63 mg QE/g EEP). The propolis samples from Greater Poland (P14) and from West Pomerania (P15) were characterized by a high total content of phenols (Figure 1A) and flavonoids (Figure 1B), while propolis from Lublin Province (P3) was characterized by the lowest values of TPC and TFC, as shown in Figure 1A and 1B, respectively. 

The values of TFC determined for propolis collected from three regions of Poland were: 18.76 mg QE/g for propolis collected from Podlaskie Province, 22.19 mg QE/g for Masovia propolis and 19.79 mg QE/g for propolis from West Pomerania [45]. In turn, total flavonoid content in Polish propolis extract determined by Socha et al. [24] was in a range from 35.64 (propolis from Podlaskie Province) to 62.04 mg QE/g (sample from Świętokrzyskie Province). Propolis collected from 20 different localizations of South Korea was characterized by TFC in a range from 20.8 to 49.8 mg QE/g EEP [47]. The TFC value in extracts of propolis harvested in Brazil was 53.0 mg QE/g EEP, sample from Australia–38.0 mg QE/g EEP, and propolis from China—32/5 mg QE/g EEP [47]. Moreover, the TFC values in extracts of propolis collected from 28 localizations of East Andalusia (southern Spain) were in a range from 60.3 to 138.3 mg QE/g EEP [50]. TFC in hydroethanolic extracts of propolis from apiaries located in different parts of Portugal was in a range from 25.15 to 142.32 mg QE/g [51]. Moreover, the results described by Silva et al. [51] indicated that hydroethanolic extracts of propolis contained higher TFC than methanolic and aqueous. The TFC values of propolis extracts from various provinces of China were in a range from 8.3 to 188 mg QE/g EEP [52]. In turn, total flavonoid content of propolis extracts collected from various counties was determined by Kumazawa et al. [53]. The highest TFC value was determined for propolis collected in Hungary (176 mg QE/g EEP), while the lowest values were found in propolis harvested from Thailand (2.5 mg QE/g EEP) [53]. 

#### 2.1.2. Profile of Phenolic Compounds

The concentration of individual phenolic compounds, including flavonoids, phenolic acids, and caffeic acid phenethyl ester (CAPE) in the extracts of propolis collected from different regions of Poland, detected by ultra-performance liquid chromatography (UPLC) are presented in Table 1. 

The examined propolis extracts were characterized by a varied content of flavonoids, both qualitatively and quantitatively. The highest total concentration of all analyzed flavonoids was determined for propolis P2 collected in Lower Silesia and amounted to 78.604 mg/g EEP. This propolis was also characterized by the highest concentration of epicatechin, which was 66.008 mg/g EEP. On the other hand, propolis P2 contained only three compounds from the tested flavonoids, apart from epicatechin, also myricetin and pinobanksin. The lowest total concentration of all analyzed flavonoids was determined for propolis P1 (3.118 mg/g EEP), propolis P3 (3.200 mg/g EEP), and propolis P14 (3.659 mg/g EEP). The propolis P1 and P3 were characterized by a low total concentration of determined flavonoids, which agrees with the low total flavonoid content (Figure 1B) analyzed in these samples. In turn, propolis P14 contained a low total concentration of analyzed flavonoids, but high total flavonoid content equals 101.22 QE/g EEP (Figure 1B), which suggests that this propolis may contain flavonoids, which was not determined in UPLC analysis. Propolis P12 was characterized by the greatest diversity of identified flavonoids—10 out of 12 analyzed flavonoids (except epicatechin and pinostrobin) were determined in a sample from Świętokrzyskie Province. The highest concentration among examined flavonoids was determined for epicatechin in propolis P2, but also propolis P4 (32.574 mg/g EEP), P5 (23.489 mg/g EEP) and P6 (42.262 mg/g EEP) contained a high amount of this compound. A high concentration was also found for pinobanksin in propolis samples: P2 (5.608 mg/g EEP), P5 (6.995 mg/g EEP), P10 (10.216 mg/g EEP), P11 (6.684 mg/g EEP), P12 (9.555 mg/g EEP), P15 (10.418 mg/g EEP), catechin in propolis samples: P8 (5.492 mg/g EEP) and P13 (6.262 mg/g EEP), and myricetin in propolis P2 (6.735 mg/g EEP) and P4 (5.223 mg/g EEP). 

Flavonoids are frequently identified in poplar-type propolis, but their profile and content varied depending on the propolis geographical origin. The flavonoid profile of the tested propolis extracts indicates that *P. nigra* may be one of the plant sources used by bees for its production. Literature data showed that *P. nigra* buds contained a variety of flavonoids, including pinocembrin, pinobanksin, chrysin, galangin, vanillin, apigenin, and pinostrobin, which were also detected in some extracts tested [16,54,55,56]. In propolis samples collected from various regions of Poland, many different flavonoids have been identified, including those determined in this study (pinocembrin, chrysin, galangin, naringenin, kaempferol, catechin, quercetin, pinobanksin, apigenin, pinostrobin, and myricetin), as well as other compounds, including sakuranetin, isorhamnetin, acacetin, apiin, luteolin, and hesperidin [1,3,4,24,28,42]. In examined propolis, a high concentration of epicatechin was determined, but in other Polish propolis samples, this flavonoid was not identified [26,42]. However, epicatechin was found in propolis from Greece, Cyprus, and Brazil [57,58]. Pinocembrin and galangin were found in high concentration in Polish propolis collected from Warmia-Masuria Province, while chrysin and pinocembrin were identified in high content in propolis harvested from various regions of the country [26,28]. The high content of chrysin and galangin was determined in Polish propolis from Lower Silesia, West Pomerania, and Subcarpathia Province, while a high concentration of chrysin and apigenin was found in propolis from Beskid Mountains [3,4]. Moreover, all tested flavonoids were identified in propolis samples from various geographical regions, including Spain, Italy China, Argentina, Ukraine, Macedonia, Croatia, and Romania [4,50,59,60,61,62]. 

The examined propolis extracts were characterized by a wide diversity of phenolic acids concentration. Syringic acid was the only acid determined in all propolis extracts in a concentration range from 0.097 (sample P7) to 2.873 mg/g EEP (sample P2). Propolis collected in Lower Silesia (P1) was characterized by the greatest diversity of identified phenolic acids—6 out of 8 analyzed compounds, except caffeic and vanillic acids, were determined in this sample. In contrast, only three phenolic acids were found in four propolis samples (P7—ferulic, 3-hydroxycinnamic, and syringic acids; P8, P9, P11—ferulic, syringic, and vanillic acids). The highest total concentration of all examined phenolic acids was found in propolis P1 from Lower Silesia (11.731 mg/g EEP), while the lowest total amount of examined phenolic acids was detected in propolis P7 from Masovia (0.296 mg/g EEP). A high concentration of coumaric acid (3.504 mg/g EEP) and 3-hydroxycinnamic acid (4.963 mg/g EEP) was found in propolis P1 from Lower Silesia. In turn, a high content of vanillic acid was determined in three propolis samples: P8 (5.812 mg/g EEP), P9 (4.264 mg/g EEP), and P10 (3.583 mg/g EEP). The aromatic acids determined in the examined propolis extracts were also detected in bee glue plant precursors—*P. nigra* and *P. tremula*. Literature reports showed that both *Populus* species were characterized by the content of such aromatic acids as caffeic, cinnamic, ferulic, and coumaric [16,54,55,56].

In propolis samples from various regions of Poland were identified phenolic acids, including those determined in this study (caffeic, cinnamic, coumaric, ferulic, syringic, 3-hydroxycinnamic and vanillic acids), as well as other acids, such as gallic, hydroxybenzoic, genistic, benzoic, dihydrocinnamic, and isoferulic [1,24,26,42,43]. Moreover, in examined propolis extracts, the presence of sinapic acid was found, which was previously identified in propolis samples from Greece, Argentina, or Brazil [58,63,64]. Socha et al. [24] found that Polish propolis contained a high concentration of coumaric acid in the range from 37.54 mg/g (sample from Kuyavian-Pomeranian Province) to 116.95 mg/g (sample from Lesser Poland). Moreover, the concentration of caffeic (7.68–20.09 mg/g) and ferulic (19.32–44.47 mg/g) acids was detected at a high level in propolis from various regions of Poland [24]. A high content of coumaric acid was detected in extracts of propolis collected from apiaries located in Warmia-Masuria Province and Greater Poland [26,42,43]. A relatively high content of vanillic acid (1.306–5.812 mg/g EEP) detected in propolis extracts (samples P8-P14) occurred for the first time in Polish propolis, so far the concentration of this acid has been determined at a much lower level [26,42,43]. The presence of the analyzed phenolic acids was confirmed previously in propolis samples from various parts of the world, including China, Italy, Hungary, Chile, and Greece [17,52,53,58,61]. 

The examined propolis extracts contained also varied concentrations of caffeic acid phenethyl ester (CAPE). The ester was detected in all propolis extracts in a concentration range from 0.035 (sample P7 from Masovia Province) to 1.014 mg/g EEP (sample P14 from Greater Poland). The presence of CAPE was previously found in the Polish propolis and samples from other countries, such as Italy, Spain, China, Ukraine, Macedonia, Australia, and the United States [50,61,62]. Italian propolis contained CAPE in a range from 2.36 to 4.27 mg/g EEP, Australian propolis in content equal to 10.4 mg/g EEP, and Bulgarian—5.6 mg/g EEP, while in propolis samples from Brazil, South Africa, and Thailand, the presence of CAPE was not confirmed [22,52,53]. 

### 2.2. Biological Activity of Propolis Extracts

The biological activity of ethanolic propolis extracts was evaluated, including antioxidant activity, effect of extracts on human red blood cells (RBCs), antifungal and antibacterial properties, as well as their antiviral potential.

#### 2.2.1. Antioxidant Activity

**Figure 2:**
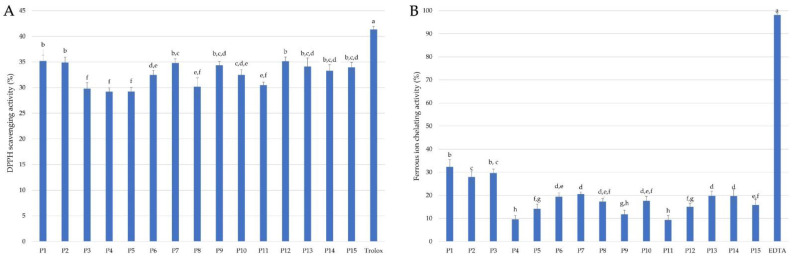
DPPH free radical scavenging activity (**A**) and ferrous ion chelating activity (**B**) of propolis extracts used at the concentration 0.1 mg/mL. (Values denoted with identical letters do not differ significantly).

The antioxidant potential of propolis extracts was assessed based on two cell-free assays—DPPH radical scavenging assay and ferrous ion chelating activity assay with ferrozine, which results are presented in Figure 2.

The propolis extracts were characterized by varied DPPH free radical scavenging activity ranging from 35.14% to 29.22%. The highest DPPH scavenging ability possessed extract of propolis P12 collected from Świętokrzyskie Province (35.14%), and two samples from Lower Silesia (P1: 35.20%, P2: 34.90%), which corresponding about 85% of Trolox activity, used in this study as a reference antioxidant. In turn, propolis samples from Łódzkie Province (P4: 29.22%) and Lesser Poland (P5: 29.24%) were characterized by the lowest values of the antiradical activity toward DPPH, which was about 70% of the activity determined for the standard antioxidant Trolox.

Propolis samples collected from different parts of the world, including Brazil, China, Spain, Italy, and Greece were characterized by various antiradical activities assessed in the DPPH radical scavenging assay [22,50,58,65]. Ethanolic and hexane–ethanol extracts of propolis collected from different regions of Poland (Masovia, West Pomerania, and Podlaskie Province) showed higher DPPH free radical scavenging activity compared to ethanol–hexane and hexane extracts [45]. Socha et al. [24] reported that the ability of propolis extracts collected from nine areas of Poland to scavenge DPPH radicals was comparable. Kumazawa et al. [53] examined the antiradical activity of 16 propolis samples collected from 14 countries using DPPH assay and found that the sample from Australia showed the highest antiradical potential, while propolis from Thailand exhibited the lowest antiradical activity. In turn, Ahn et al. [52] reported that the antiradical activity of Chinese propolis was related to the region of the country where it was harvested. 

The examined propolis extracts exhibited relatively low ferrous iron chelating activity ranging from 9.33% to 32.32%, which corresponds to 9.5% and 32.9% of EDTA (ethylenediaminetetraacetic acid) activity, used as the ferrous ions standard chelator. The highest activity of Fe^2+^-chelating activity possessed propolis P3 from Lublin Province (29.63%) and two samples from Lower Silesia (P1: 32.32% and P2: 27.91%), while the lowest activity exhibited propolis P4 from Łódzkie Province (9.62%) and sample from Pomerania Province (9.33%). The tested propolis extracts showed comparable chelating activity to methanolic extracts (4.33–29.68%) of propolis from Portugal and significantly lower than aqueous extracts (41.11–82.35%) of this propolis [49]. Gülçin et al. [66] indicated that lyophilized aqueous extract of propolis from Turkey showed a strong chelating effect on Fe^2+^ ions, higher than Trolox, but lower than other standards: α-tocopherol, EDTA, BHA, and BHT. Moreover, extracts of propolis from two provinces of Poland (Greater Poland and Warmia-Masuria Province) were characterized by a higher ferrous ion chelating effect than all tested extracts [26,42]. 

#### 2.2.2. Effect of Propolis Extracts on Human Red Blood Cells (RBCs) Membrane under In Vitro Physiological and Oxidative Stress Conditions

**Figure 3:**
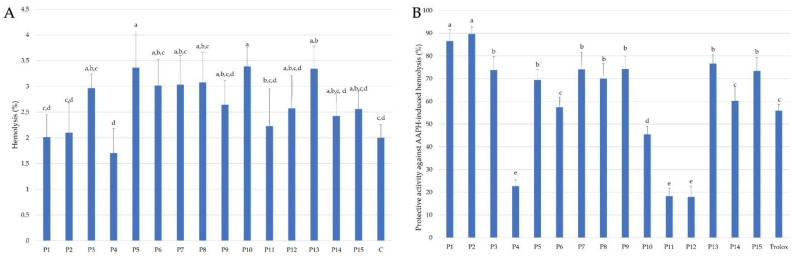
Hemolytic activity (**A**) and cytoprotective effects (**B**) of propolis extracts used at the concentration 0.05 mg/mL against 2,2’-azobis(2-methylpropionamidine) dihydrochloride (AAPH)-induced oxidative hemolysis (means ± SD). (Values denoted with identical letters do not differ significantly).

Hemolytic assay in vitro is the most important assay in the evaluation of RBC membrane-perturbing activity of bioactive compounds proposed for any biomedical applications [67]. The hemolytic activity of any bioactive compounds or extracts higher than 5%, excludes them from further in vitro evaluation and in vivo biomedical applications at a given hemolytic concentration [26,42,43]. As shown in Figure 3A, the hemolytic activity of all propolis extracts used in this study at a concentration equal to 0.05 mg/mL, was lower than 5% (<3.5%); therefore, it can be concluded that extracts of propolis harvested in different regions in Poland are biocompatible agents without any detrimental effects on the molecular structure of the RBC membrane and can be proposed for furthers assessment of their cytoprotective activity under oxidative stress conditions.

In our previous study, it was shown that different propolis extracts protect RBCs against free-radical induced hemolysis dependent on the type of solvent used for the extraction process and the chemical compositions, mostly phenolic compounds content [26,42,43]. As shown in Figure 3B, the ethanolic extracts of Polish propolis protect human RBCs against free radicals generated from AAPH in the range from about 22% (P4, P11, P12) to about 90% (P1 and P2). Moreover, 9 (P1–P3, P5, P7–P9, P13, P15) out of 15 tested propolis extracts showed significantly higher cytoprotective activity than Trolox (55%), used as a reference antioxidant. Propolis samples P1 and P2, both collected in the region of Lower Silesia but from a different city, were the most effective in DPPH radical scavenging (Figure 2A) and sample P1 was the best ferrous ions chelator (Figure 2B). The highest antioxidant activity, as well as cytoprotective properties of propolis extract P2, can be explained by the highest total concentration of all analyzed flavonoids equal to 78.604 mg/g EEP (Table 1) with epicatechin as the most represented flavonoid. On the other hand, propolis extract P1 has the lowest total concentration of all analyzed flavonoids (3.118 mg/g EEP). Therefore, the total concentration of flavonoids in the propolis extracts is not sufficient for simple prediction and explanation of their cytoprotective properties. Apart from the antioxidant potential confirmed in the DPPH assay and ferrous ions assay, specific interactions of numerous compounds in the propolis extracts with RBC cell membrane are indicated to explain the high cytoprotective activity of propolis P1 and P2. 

#### 2.2.3. Antibacterial and Antifungal Activity

The activity of ethanolic extracts of propolis collected from 15 regions of Poland against bacterial and fungal strains is presented in Table 2.

The examined propolis extracts showed various activity against the tested bacterial strains, both Gram-positive and Gram-negative. The most sensitive bacterial strain against all extracts was *B. cereus*, and the propolis extracts from Greater Poland (P14) and West Pomerania (P15) gave the inhibition zone equal to 25 and 27 mm, respectively. All extracts inhibited moderate activity against *P. aeruginosa* and *K. pneumonia*, with the exception of the propolis extract (P4) from Łódzkie Province, which showed high activity against these bacterial strains, and the propolis extract (P10) from Pomerania, which was characterized by high activity against *P. aeruginosa*. The activity against *S. enteritidis* of propolis extracts varied and related to the place of propolis collection—propolis samples P1–P6 showed low activity, while samples P11 and P14 exhibited high antibacterial activity and the rest of the propolis samples were characterized by moderate activity. Moreover, the activity of propolis extracts against *L. monocytogenes* varied greatly depending on the origin of the propolis. All propolis extracts showed low activity against *E. coli* and were inactive against *S. aureus*. Moreover, all propolis extracts have not shown activity against probiotic bacteria—*Lb. paracesei.* Additionally, most of the tested propolis extracts (samples P1–P10) showed no activity against *S. cerevisiae*. Four of the samples exhibited moderate activity (samples P11–P14), and only one sample (P15) from West Pomerania was characterized by high activity against this yeast strain. In turn, part of the propolis extracts (samples P1–P3, P6) showed no activity against *C. albicans*, samples P7 and P8 exhibited low activity, samples P9–P13 had moderate activity, and extracts prepared from propolis samples P4, P5, P14, and P15 were characterized by high activity against this pathogenic fungus. The obtained results showed that in propolis samples collected in different locations, different combinations of bioactive components may be responsible for the antimicrobial activity of propolis extracts.

Literature data reports that extracts of propolis collected from various geographical origin were characterized by a diversity of antifungal and antibacterial properties [32,68,69,70]. Polish propolis showed activity against various strains of bacteria, including both Gram-positive (*S. aureus*, *Staphylococcus epidermidis*, *Bacillus subtilis*, *Micrococcus luteus*, *Bacillus megaterium*, *Bacillus brevis*, *Enterococcus faecalis*) and Gram-negative bacteria (*E. coli*, *Enterobacter cloacae*, *K. pneumoniae*, *P. aeruginosa*, *Pseudomonas syringae, Proteus mirabilis, Citrobacter freundii*) [1,29,40,69]. The research of Popova et al. [1] indicated that the dichloromethane extract of Polish propolis showed promising antibacterial activity and was higher than the methanolic extract. In turn, research of Wojtyczka et al. [71] showed that the ethanolic extract of Polish propolis showed activity against methicillin-sensitive *S. aureus* and methicillin-resistant *S. aureus* clinical isolates, with minimal inhibitory concentration (MIC) within the range from 0.39 to 0.78 mg/mL. Extracts of propolis collected from various parts of Poland also showed activity of fungal strains, including *C. albicans*, *Candida tropicalis*, *Candida glabrata*, *Trichoderma viride*, *Aspergillus niger*, *Penicillium chrysogenum*, *S. cerevisiae*, *Aspergillus versicolor*, *Paecilomyces variotii*, *Penicillium funiculosum*, *Penicillium cyclopium,* and *Aureobasidium pullulans* [1,26,29,69]. Moreover, the results described in the literature indicated that Polish propolis showed higher activity against bacteria than fungi [1,29,69]. 

#### 2.2.4. Antiviral Activity

**Figure 4:**
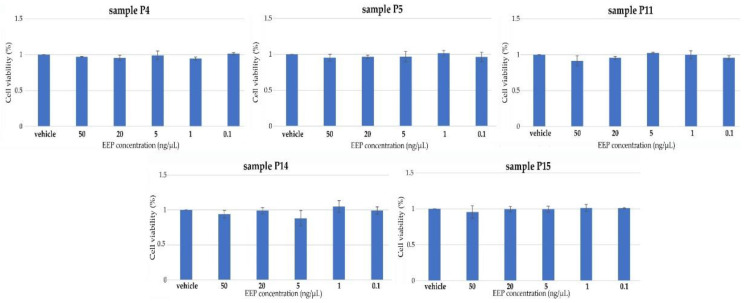
The effect of selected propolis extracts (samples P4, P5, P11, P15, and P15) on HaCaT cell viability. The data are expressed as the mean viability from three independent experiments in each case. (All values in graphs were statistically insignificant).

**Figure 5:**
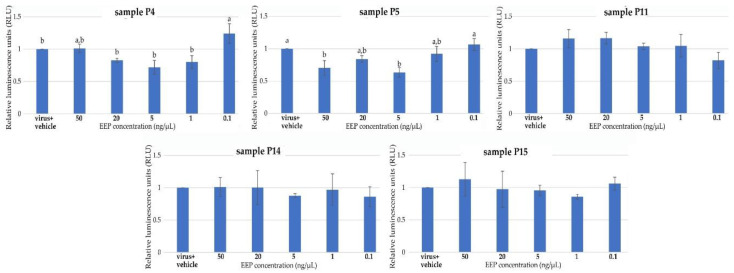
The effect of selected propolis extracts (samples P4, P5, P11, P15, and P15) on HPV16 (PsVs) infection. The data are expressed as the mean viability from three independent experiments in each case. (Values denoted with identical letters do not differ significantly; values without letters in graphs were statistically insignificant).

The antiviral activity of the selected propolis extracts (samples P4, P5, P11, P14, and P15) was evaluated using HPV16 (human papillomaviruses) pseudovirions (PsVs) system and HaCaT cells and the results are presented in Figure 4 and Figure 5.

In the first stage of research on the antiviral activity of propolis, the effect of propolis extracts on the viability of HaCaT cells was evaluated. Cytotoxicity studies showed that all selected propolis extracts (samples P4, P5, P11, P14, and P15) are very toxic for HaCaT at high concentrations (10 mg/mL, 5 mg/mL, 1 mg/mL, 100 ng/µL). At the same time, cell viability was not affected by any of the selected propolis extracts at lower concentrations (50 ng/µL, 20 ng/µL, 5 ng/µL, 1 ng/µL, 0.1 ng/µL), which confirmed by statistical analysis (data not shown). 

To analyze the effect of propolis extracts on HPV16 PsVs infectivity in HaCaT cells, all selected samples at non-toxic concentrations (50 ng/µL, 20 ng/µL, 5 ng/µL, 1 ng/µL, 0.1 ng/µL) were tested. Infectious assays showed that the examined propolis extracts have various activities against the tested HPV16 PsVs. The statistical analysis indicated significant differences in the concentrations of propolis extracts from Łódzkie Province (P4) and Lesser Poland (P5) in terms of their ability to reduce HPV16 infection (PsVs), as shown in Figure 5. The propolis extracts from Łódzkie Province (P4) and Lesser Poland (P5) reduce HPV16 infection by around 30% and 40%, respectively, at 5 ng/µL concentration. Propolis extracts from Pomerania (P11), Greater Poland (P14), and West Pomerania (P15) did not affect HPV16 PsVs infectivity (Figure 5).

Despite intensive studies on the antiviral activity of propolis on different DNA and RNA viruses [2,72,73], its effect on HPV has not yet been studied. All obtained results suggest that propolis has moderate activity against HPV and confirms that the antiviral activity of propolis extracts depends on geographical distribution. However, more studies are needed to elucidate the mechanisms of propolis action and identify the components responsible for the antiviral effects of this natural product.

## 3. Materials and Methods

### 3.1. Propolis Samples and Preparation of Propolis Extracts

Raw propolis samples were collected from apiaries localized in 15 various regions of Poland and purchased from a beekeeping company (PROKIT, Halinów, Poland). The localizations of apiaries are presented in Table 3. 

The ground propolis samples (15 g) were extracted with 150 mL of 70% ethanol (Avantor Performance Materials, Gliwice, Poland) under shaking (Biosan, Riga, Latvia) for 5 days in the dark and at ambient temperature. After extraction, propolis solutions were filtered through a Whatman no. 4 filter paper (Sigma-Aldrich, Steinheim, Germany) and evaporated using a rotary evaporator (Buchi Labortechnik AG, Flawil, Switzerland) to constant weight. The yield of the extraction process was evaluated by comparing the dry weight of extract (residue) with the initial weight of propolis, using Equation (1):

(1) $$\mathrm{Yield}\;(\%)=\frac{W_{propolis\;extract\;residue}}{W_{propolis}\;}\;\cdot 100\%$$

where *W_propolis extract residue_* (g) is the weight of propolis after solvent evaporation and *W_propolis_* (g) is the weight of the initial dried propolis. 

The extraction yield of propolis samples is presented in Table 3. The obtained residues of propolis extracts were next used in biological and chemical analyses. 

### 3.2. Determination of Total Phenolic Content (TPC)

The total phenolic content of propolis extracts was determined using the Folin-Ciocalteu procedure [74]. The ethanolic extract of propolis (0.1 mL) at 0.5 mg/mL concentration was reacted with 0.25 mL of Folin–Ciocalteu reagent (Sigma-Aldrich, Steinheim, Germany) for 3 min. Then, 3 mL Na_2_CO_3_ solution (Avantor Performance Materials, Gliwice, Poland) at 10% (*w*/*v*) concentration was added to the reaction mixture. The absorbance readings were taken at 765 nm using a Cary 300 Bio UV-Visible scanning spectrophotometer (Agilent Technologies, Santa Clara, CA, USA) after incubation for 40 min in the dark and at ambient temperature. Results were expressed as gallic acid equivalent (mg gallic acid (GA)/g dry weight of ethanolic extract of propolis (EEP)—mg GAE/g EEP). All measurements were made in triplicate.

### 3.3. Determination of Total Flavonoid Content (TFC)

The total flavonoid content of propolis extracts was determined using the aluminum chloride colorimetric method based on the procedure described by Ahn et al. [52]. The ethanolic extract of propolis (0.2 mL) at 0.5 mg/mL concertation was reacted with 2 mL of 2% (*w*/*v*) AlCl_3_ solution (Avantor Performance Materials, Gliwice, Poland). The absorbance was read at 430 nm using a Cary 300 Bio UV-Visible scanning spectrophotometer (Agilent Technologies, Santa Clara, CA, USA) after 1 h incubation in the dark and at ambient temperature. Results were expressed as mg of quercetin equivalent in g of ethanolic propolis extract (mg QE/g EEP). All measurements were made in triplicate.

### 3.4. Phenolic Profile of Propolis Extracts

The concentration of flavonoids (apigenin, catechin, epicatechin, chrysin, myricetin, pinocembrin, pinobanksin, pinostrobin, naringenin, quercetin, galangin, kaempferol), phenolic acids (caffeic, ferulic, vanillic, syringic, sinapic, coumaric, 3-hydroxycinnamic, and cinnamic) and caffeic acid phenethyl ester (CAPE) in propolis extracts was determined using the Aquity UPLC chromatograph (Waters, Manchester, MA, USA) equipped with a photodiode detector (PDA eλ Detector) (Waters, Manchester, MA, USA) and coupled to an electrospray ionization triple-quadrupole mass spectrometer (TQD) (Waters, Manchester, MA, USA) according to the previously described method [26]. Detailed identification parameters of phenolic compounds for the UPLC/PDA/TQD method are presented in Supplementary Materials (Supplementary Materials: Figure S1 and Table S1). The standard and solvents used in the chromatographic analysis were purchased from Sigma-Aldrich (Steinheim, Germany). The initial concentration of each standard in methanol was 100 µg/mL. The residues of propolis extracts (0.5 g) were dissolved in methanol (2 mL) and filtered through a 0.20 µm syringe filter (Chromafil, Macherey-Nagel, Duren, Germany) before analyses. The phenolic compounds were separated at 25 °C on a Waters ACQUITY UPLC HSS T3 column (150 × 2.1 mm/ID, with 1.8 µm particle size) (Waters, Manchester, MA, USA) and applying the Empower^TM^ 3 software (Waters, Milford MA, USA). Gradient elution was applied using water containing 0.1% HCOOH (A) and acetonitrile containing 0.1% HCOOH (B) with a flow rate of 0.3 mL/min. The solvent gradient was modified as follows: 0–5 min 25% B, 5–20 min 40% B, 20–30 min 60% B, 30–35 min 90% B, and 35–40 min 100% B, followed by a return to the initial conditions. The injection volume was 3 µL. The mass spectrometer was operated in the negative or positive-ion mode with the full scan in the mass range from m/z 100 to 600. The results are presented as the average of three measurements.

### 3.5. Antioxidant Activity of Propolis Extracts

The antioxidant activity of the propolis extracts was assessed on the basis of cell-free assays: free-radical scavenging activity and ferrous ion chelating activity, according to the previously described method [26]. The concentration of ethanolic extract of propolis used in these assays was 0.1 mg/mL.

The ethanolic solution (0.2 mL) of DPPH· (2,2-diphenyl-1-picrylhydrazyl, Sigma-Aldrich, Steinheim, Germany) at 0.1 mM concentration was added to 0.2 mL of propolis extracts and vortexed (Bio Vortex V1, Biosan, Riga, Latvia). The extracts and reference compounds (Trolox, Sigma-Aldrich, Steinheim, Germany) were incubated for 30 min in the dark and at ambient temperature. The absorbance (Abs) was measured at 517 nm using a BioMate™ 160 UV-Visible spectrophotometer (Thermo Scientific, Waltham, MA, USA). The percentage DPPH· scavenging effect was calculated according to the following equation:

(2) $$DPPH\cdot scavenging\;activity\;\left(\%\right)=\frac{Abs_{0\;}-Abs_{1\;}\;}{Abs_{0}}\cdot 100\%$$

where *Abs_0_*—absorbance of the control samples; *Abs_1_*—absorbance of the propolis extract. Every tested sample was prepared in triplicate and every experiment was repeated three times. The results (n = 9) are presented as a mean value ± standard deviation (±SD). 

The propolis extracts (0.2 mL) or reference compound (EDTA—ethylenediaminetetraacetic acid, Sigma-Aldrich, Steinheim, Germany) were mixed with a 0.05 mL solution of chloride irons (Sigma-Aldrich, Steinheim, Germany) at a 0.6 mM concentration. The reaction was started by the addition of 0.05 mL ethanolic solution of ferrozine (Sigma-Aldrich, Steinheim, Germany) at a 5 mM concentration. The mixture was immediately shaken (Bio Vortex V1, Biosan, Riga, Latvia), then stored for 10 min at ambient temperature. The absorbance (Abs) of tested solutions was measured at 562 nm using a BioMate™ 160 UV-Visible spectrophotometer (Thermo Scientific, Waltham, MA, USA). The percentage of inhibition of ferrozine-Fe^2+^ complex formation was calculated according to the following equation:

(3) $$Fe^{2+}chelating\;effect\;\left(\%\right)=\left[1-\frac{Abs_{1\;}\;}{Abs_{0}}\right]\cdot 100\%$$

where *Abs_0_*—absorbance of the control samples; *Abs_1_*—absorbance of the propolis extract. Every tested sample was prepared in triplicate and every experiment was repeated three times. The results (n = 9) are presented as a mean value ± standard deviation (±SD). 

### 3.6. Hemolytic Activity of Propolis Extracts

Fresh human red blood cells (RBCs) concentrates (hematocrit = 65%) were purchased from the blood bank according to the bilateral agreement no. ZP/907/1002/18 signed between the Regional Blood Center in Poznań and Adam Mickiewicz University. Hemolytic activity of propolis extracts was evaluated using RBCs, as previously reported [42]. Briefly, RBCs (1.65 × 10^8^ cells/mL, hematocrit 1.5%) were incubated in PBS (7.4 pH) supplemented with 10 mM glucose and containing extracts tested at a concentration equal to 0.05 mg/mL for 60 min at 37 °C under gentle shaking. Samples with RBCs incubated in PBS without extracts tested were taken as the control. Each sample was prepared in triplicate and the experiments were repeated three times with RBCs from different donors. After incubation, RBC suspensions were centrifuged (3000 rpm, 10 min, +4 °C), and the degree of hemolysis was estimated by measuring the absorbance of the supernatant at λ = 540 nm in a BioMate™ 160 UV-Vis spectrophotometer (Thermo Scientific, Waltham, MA, USA). The results were expressed as a percentage (%) of hemolysis. Hemolysis 0% was taken as the absorbance of the supernatant of RBC suspensions in PBS buffer, while the total hemolysis (100%) was determined when PBS was replaced by ice-cold distilled water. A hemolysis degree < 5% indicates no hemolytic activity of an extract studied. The results are presented as a mean value (±SD) of three independent experiments.

### 3.7. Inhibition of Oxidative Stress-Induced Hemolysis of Human RBC by Propolis Extracts

The cytoprotective activity of propolis extracts was evaluated according to the previously described method [42]. Briefly, RBCs (1.65 × 10^8^ cells/mL, 1.5% hematocrit) were preincubated in PBS (pH 7.4) supplemented with 10 mM glucose and containing propolis extracts tested at a concentration of 0.05 mg/mL for 20 min at 37 °C under gentle shaking. Trolox was used as the standard antioxidant at the same concentration. After pre-incubation with propolis extracts, 2,2′-azobis(2-methylpropionamidine) dihydrochloride (AAPH) was added at a final concentration of 60 mM, and samples were incubated for the next four hours. RBCs incubated in PBS, and the presence of AAPH, were taken as the negative and positive controls, respectively. After incubation, the RBC suspensions were centrifuged (3000 rpm, 5 min, +4 °C), and the degree of hemolysis was determined by measuring the absorbance (Abs) of the supernatant at λ = 540 nm in BioMate™ 160 UV-Vis spectrophotometer (Thermo Scientific, Waltham, MA, USA). The percentage of AAPH-induced hemolysis inhibition was calculated using the following equation:

(4) $$\;\;\;\;\;\;\;Inhibition\;of\;hemolysis\;\left(\%\right)=100-\left[\left(\frac{Abs_{pex\;}-Abs_{PBS\;}\;}{Abs_{AAPH}-\;Abs_{PBS}}\right)\cdot 100\%\right]$$

where *Abs_pex_*—the absorbance value of supernatant obtained from samples incubated with propolis extracts in the presence of AAPH, *Abs_PBS_*—the absorbance of the supernatant obtained from PBS controls, and *Abs_AAPH_*—the absorbance of the supernatant obtained from AAPH controls. Each sample was made in triplicate, and the results are presented as a mean value (±SD) of three independent experiments with RBCs obtained from different donors.

### 3.8. Antibacterial and Antifungal Activity of Propolis Extracts

The antibacterial and antifungal activity of propolis extracts at 100 mg/mL concentration was determined using the agar diffusion method.

The pathogenic bacteria strains: *Listeria monocytogenes* (ATCC 15313), *Staphylococcus aureus* (ATCC 25923), *Bacillus cereus* (ATCC 10876), *Escherichia coli* (ATCC 10536), *Pseudomonas aeruginosa* (ATCC 15443), *Salmonella* Enteritidis (05/07 isolate from the collection of the Department of Biotechnology and Food Microbiology), *Klebsiella pneumoniae* (895 isolates from the collection of the Department of Biotechnology and Food Microbiology), probiotic bacteria strain *Lacticaseibacillus paracasei* (CNCM I-1572) and fungal strains: *Candida albicans* (ATCC 10231) and *Saccharomyces cerevisiae* (ATCC 9763) were used in the assays. All indicator strains were stored in a Cryobank (Bacteria storage system, MAST Diagnostica) at −20 °C. Prior to the tests, the strains were defrosted and passaged twice in nutrient broth (OXOID CM 0001) with the addition of 2% (*w*/*v*) glucose. All strains were grown at 35 ± 2 °C for 24 h. To determine the antimicrobial activity, Muller–Hinton agar (OXOID CM 0337) was applied. The Petri dishes with this medium were inoculated with a standardized inoculum of indicator strains at the level 10^5^ CFU/mL. Next, 10 µL of each tested sample was spotted on the agar surface. The plates prepared in this way were then incubated at 35 °C ± 2 °C for 24 h. After this time, the diameters of inhibition growth zones were measured using a Computer Scanning System (MultiScaneBase v14.02). The results are expressed in millimeters. 

### 3.9. Antiviral Activity of Propolis Extracts

The antiviral activity of selected propolis extracts (samples P4, P5, P11, P14, and P15) was evaluated using the HPV-16 (human papillomaviruses) pseudovirions (PsVs) system. 

HPV type 16 Pseudovirions packaged with a pGL3 Luci construct expressing firefly luciferase were produced in HEK 293 TT cells by the method developed by Buck et al. [75]. Purity and capsid protein content was determined by SDS-PAGE and Coomassie brilliant blue staining; the encapsidated DNA was analyzed by real-time PCR, and the copy number was quantified using a standard curve of reporter plasmid DNA. 

HPV infectivity in the presence of selected propolis extracts was analyzed using luciferase assay, and HaCaT cells (the immortalized human keratinocytes) as described previously [76]. First, the viability of HaCaT cells treated with selected propolis extract at different concentrations was assessed using a trypan blue exclusion assay as described before [77]. At 36 h after propolis treatment, the cells were trypsinized, collected, and stained with 0.2% trypan blue. The number of viable cells in samples treated with different propolis concentrations was normalized to the number of viable cells obtained for the vehicle (1% ethanol)—treated samples. Next, HaCaT cells were exposed for 1 h at 37 °C to selected propolis extracts at the maximum non-cytotoxic concentrations, and then 100 viral genome equivalents (vge)/cell of HPV-16 PsVs were added. The HPV infection was monitored after 36 h by luminometric analysis of firefly luciferase activity using a luciferase assay system (Promega, Madison, Wisconsin, USA). For each sample, the infection was normalized to 100% as the value obtained with cells treated with vehicle (1% ethanol). This was used to calculate the percentage reduction following treatment of the cells with different concentrations of propolis. Equal amounts of total cell protein extracts were used in the luciferase measurements.

### 3.10. Statistical Analysis

Statistical analyses included factorial one-way ANOVA, followed by Tukey’s honest significant difference (HSD) test at α = 0.05. All statistical analyses were performed using the TIBCO Software Inc. Statistica version 13.3 (Palo Alto, CA, USA). 

## 4. Conclusions

The paper presents the results of the chemical composition and biological activity of propolis samples collected from 15 apiaries located in various parts of Poland. 

The examined propolis extracts were characterized by different total phenolic content (116.16–219.41 mg GAE/g EEP) and total flavonoid content (29.63–106.07 mg QE/g EEP) depending on the place of propolis harvest. The chromatographic analysis of the phenolic profile indicated that propolis extract samples were characterized by a varied content of flavonoids and phenolic acids, both qualitatively and quantitatively. A high concentration of epicatechin, catechin, pinobanksin, myricetin, and acids: syringic and vanillic was detected in propolis extracts. The presence of caffeic acid phenethyl ester was confirmed in all tested propolis extracts, and the CAPE content was in the range from 0.035 to 1.014 mg/g EEP. 

The location of propolis sampling influenced the biological properties of their extracts. The ethanolic propolis extracts were characterized by moderate DPPH free radical scavenging activity, ranging from 29.22% to 35.14%, and relatively low ferrous iron chelating activity ranging from 9.33% to 32.32%. The results indicated also that the propolis extracts protect human RBCs against free radicals generated from AAPH in the range of 22.69% to 89.65%. Moreover, 9 out of 15 examined extracts possessed cytoprotective activity significantly higher than Trolox, used as the reference antioxidant. The propolis extracts showed various activity against the tested pathogenic bacterial and fungal strains. The most sensitive bacterial strain against all extracts was *B. cereus*, with an inhibition zone ranging from 12 mm to 27 mm. In turn, all extracts exhibited low activity against *E. coli*, and were inactive against *S. aureus*. Moreover, all propolis extracts did not show activity against probiotic bacteria—*Lb. paracesei.* The activity of propolis against both tested fungal strains (*S. cerevisiae* and *C. albicans*) was limited. The results of the antimicrobial properties of propolis extracts indicated that activity against bacteria, fungi, and viruses depended on propolis origin. The research on the antiviral effect of selected propolis extracts indicated that only 2 of 5 examined samples showed moderate activity against HPV and this activity depended on its geographical distribution. 

It can be concluded that the chemical composition and biological properties of propolis are related to its geographical origin. Even samples of propolis from the same country differ in chemical composition and biological properties. Therefore, further research on the chemical composition and biological activity of propolis from various parts of the world is important in terms of its standardization, and thus its safe use in many applications. Moreover, the results indicated that in different propolis samples different bioactive compound combinations are essential for the biological activity of its extracts. 

## Figures and Tables

**Table 1:** The concentration of phenolic compounds in propolis extracts.

Concentration (mg/g EEP)	Propolis Samples														
	P1	P2	P3	P4	P5	P6	P7	P8	P9	P10	P11	P12	P13	P14	P15
Apigenin	nd	nd	0.328 ^c^ ± 0.019	nd	1.194 ^a^ ± 0.073	0.145 ^d^ ± 0.010	nd	nd	nd	0.106 ^d,e^ ± 0.013	0.386 ^c^ ± 0.022	0.123 ^d,e^ ± 0.011	0.056 ^e,f^ ± 0.008	nd	0.477 ^b^ ± 0.033
Catechin	0.214 ^h^ ± 0.011	nd	nd	nd	Nd	0.029 ^h^ ± 0.002	3.049 ^e^ ± 0.085	5.492 ^b^ ± 0.031	4.313 ^c^ ± 0.037	2.986 ^e^ ± 0.138	3.052 ^e^ ± 0.156	3.751 ^d^ ± 0.154	6.262 ^a^ ± 0.170	1.479 ^g^ ± 0.130	2.077 ^f^ ± 0.103
Chrysin	nd	nd	nd	nd	Nd	nd	0.047 ± 0.004	nd	0.060 ± 0.008	nd	nd	0.179 ± 0.018	nd	nd	nd
Epicatechin	nd	66.008 ^a^ ± 2.928	nd	32.574 ^c^ ± 3.010	23.489 ^d^ ± 1.820	42.262 ^b^ ± 3.130	2.999 ^e,f^ ± 0.140	nd	nd	nd	nd	nd	6.578 ^e^ ± 0.191	nd	nd
Galangin	nd	nd	0.360 ^c^ ± 0.034	nd	0.034 ^f^ ± 0.004	nd	0.550 ^a^ ± 0.034	0.111 ^e^ ± 0.012	0.206 ^d^ ± 0.016	0.115 ^e^ ± 0.012	nd	0.093 ^e^ ± 0.007	nd	0.135 ^e^ ± 0.012	0.475 ^b^ ± 0.021
Kaempferol	nd	nd	0.273 ^d^ ± 0.025	nd	0.282 ^d^ ± 0.027	nd	nd	0.317 ^c,d^ ± 0.019	nd	0.075 ^e^ ± 0.004	0.468 ^b^ ± 0.032	0.258 ^d^ ± 0.014	0.286 ^d^ ± 0.015	0.389 ^c^ ± 0.019	0.840 ^a^ ± 0.048
Myricetin	0.192 ^h^ ± 0.012	6.735 ^a^ ± 0.371	1.363 ^f,g^ ± 0.092	5.223 ^b^ ± 0.224	1.964 ^d,e^ ± 0.103	3.302 ^c^ ± 0.238	3.868 ^c^ ± 0.102	nd	3.293 ^c^ ± 0.150	1.562 ^e,f^ ± 0.125	0.972 ^g^ ± 0.092	0.349 ^h^ ± 0.032	2.339 ^d^ ± 0.215	nd	nd
Naringenin	nd	nd	0.561 ^d^ ± 0.033	0.769 ^b^ ± 0.036	0.955 ^a^ ± 0.084	nd	0.642 ^c^ ± 0.038	nd	0.574 ^c,d^ ± 0.031	0.331 ^e^ ± 0.035	0.669 ^b,c^ ± 0.043	0.058 ^f,g^ ± 0.008	nd	0.130 ^f^ ± 0.004	nd
Pinobanksin	1.983 ^d^ ± 0.182	5.608 ^b^ ± 0.352	nd	nd	6.995 ^b^ ± 0.336	0.362 ^e^ ± 0.033	0.286 ^e^ ± 0.024	2.109 ^c,d^ ± 0.341	2.857 ^c,d^ ± 0.115	10.216 ^a^ ± 0.801	6.684 ^b^ ± 0.338	9.555 ^a^ ± 0.616	3.412 ^c^ ± 0.384	0.175 ^e^ ± 0.028	10.418 ^a^ ± 0.672
Pinocembrin	nd	0.253 ^b^ ± 0.026	nd	nd	Nd	nd	nd	0.493 ^a^ ± 0.029	0.022 ^f^ ± 0.002	nd	nd	0.137 ^d^ ± 0.010	0.085 ^e^ ± 0.007	nd	0.212 ^c^ ± 0.014
Pinostrobin	nd	nd	0.284 ^c^ ± 0.016	0.652 ^a^ ± 0.037	Nd	nd	nd	nd	nd	0.609 ^a,b^ ± 0.021	0.585 ^b^ ± 0.015	nd	nd	0.591 ^b^ ± 0.014	nd
Quercetin	0.729 ^a^ ± 0.037	nd	0.031 ^e,f^ ± 0.002	nd	Nd	nd	nd	nd	0.031 ^e,f^ ± 0.005	0.097 ^e^ ± 0.014	0.486 ^b^ ± 0.045	0.348 ^c^ ± 0.035	0.191 ^d^ ± 0.017	0.760 ^a^ ± 0.04	0.102 ^e^ ± 0.013
*Sum of flavonoids*	*3.118*	*78.604*	*3.200*	*39.218*	*34.913*	*46.100*	*11.441*	*8.522*	*11.356*	*16.097*	*13.302*	*14.851*	*19.209*	*3.659*	*14.601*
Caffeic acid	nd	0.048 ^a^ ± 0.003	nd	nd	Nd	nd	nd	nd	nd	0.003 ^b^ ± 0.001	nd	nd	nd	nd	nd
Cinnamic acid	0.029 ^d,e^ ± 0.003	nd	nd	nd	0.182 ^c^ ± 0.014	0.037 ^d^ ± 0.003	nd	nd	nd	nd	nd	nd	0.422 ^a^ ± 0.025	0.038 ^d^ ± 0.002	0.235 ^b^ ± 0.017
Coumaric acid	3.504 ^a^ ± 0.159	nd	0.102 ^b^ ± 0.007	0.024 ^b^ ± 0.003	0.020 ^b^ ± 0.002	0.078 ^b^ ± 0.003	nd	nd	nd	nd	nd	nd	nd	nd	nd
3-hydroxycinnamic	4.963 ^a^ ± 0.188	0.331 ^b^ ± 0.028	0.015 ^c^ ± 0.002	nd	Nd	nd	0.004 ^c^ ± 0.001	nd	nd	0.022 ^c^ ± 0.002	nd	nd	nd	nd	nd
Ferulic acid	0.956 ^b^ ± 0.068	nd	nd	0.019 ^f^ ± 0.002	Nd	nd	0.135 ^e,f^ ± 0.010	0.021 ^f^ ± 0.002	0.169 ^d,e^ ± 0.024	0.344 ^c^ ± 0.033	0.077 ^e,f^ ± 0.008	0.123 ^e,f^ ± 0.011	0.159 ^d,e^ ± 0.022	1.848 ^a^ ± 0.115	0.280 ^c,d^ ± 0.018
Syringic acid	2.122 ^b^ ± 0.108	2.873 ^a^ ± 0.112	2.143 ^b^ ± 0.113	0.599 ^d^ ± 0.009	0.787 ^c^ ± 0.011	0.591 ^d^ ± 0.009	0.157 ^g,h^ ± 0.005	0.097 ^h^ ± 0.011	0.150 ^g,h^ ± 0.003	0.279 ^f,g,h^ ± 0.017	0.439 ^d,e,f^ ± 0.008	0.126 ^h^± 0.006	0.099 ^h^ ± 0.002	0.318 ^e,f,g^ ± 0.026	0.477 ^d,e^ ± 0.020
Sinapic acid	0.157 ^b^ ± 0.004	0.073 ^c,d^ ± 0.003	0.034 ^c,d,e^ ± 0.004	0.031 ^d,e^ ± 0.001	0.103 ^b,c^ ± 0.005	nd	nd	nd	nd	nd	nd	0.014 ^d,e^ ± 0.002	nd	nd	2.909 ^a^ ± 0.074
Vanillic acid	nd	0.304 ^g,h^ ± 0.009	0.236 ^g,h^ ± 0.012	0.055 ^h^ ± 0.003	Nd	0.221 ^g,h^ ± 0.005	nd	5.812 ^a^ ± 0.137	4.264 ^b^ ± 0.180	3.583 ^c^ ± 0.194	1.306 ^f^ ± 0.204	2.003 ^e^ ± 0.133	2.808 ^d^ ± 0.196	1.446 ^f^ ± 0.040	0.567 ^g^ ± 0.010
*Sum of phenolic acid*	*11.731*	*3.629*	*2.530*	*0.728*	*1.092*	*0.927*	*0.296*	*5.930*	*4.583*	*4.231*	*1.822*	*2.266*	*3.488*	*3.650*	*4.468*
CAPE	0.983 ^a^ ± 0.053	0.878 ^a^ ± 0.049	0.198 ^d,e,f^ ± 0.023	0.211 ^d,e,f^ ± 0.017	0.554 ^b^ ± 0.026	0.848 ^a^ ± 0.046	0.035 ^e,f^ ± 0.004	0.310 ^c,d^ ± 0.027	0.228 ^d,e^ ± 0.014	0.979 ^a^ ± 0.097	0.467 ^b,c^ ± 0.022	0.213 ^d,e,f^ ± 0.025	0.319 ^c,d^ ± 0.026	1.014 ^a^ ± 0.120	0.056 ^e,f^ ± 0.013

Expressed as average ± standard deviations. Values denoted with identical letters do not differ significantly; nd—the content below detection limit of UPLC.

**Table 2:** The antimicrobial activity of propolis extracts.

Microbial Strain	Zone of Inhibition (mm)														
	P1	P2	P3	P4	P5	P6	P7	P8	P9	P10	P11	P12	P13	P14	P15
*Listeria monocytogenes*	0	0	0	0	0	0	12	12	12	10	10	12	10	10	10
*Staphylococcus aureus*	0	0	0	0	0	0	0	0	0	0	0	0	0	0	0
*Bacillus cereus*	15	17	15	20	18	14	15	15	15	15	24	12	13	25	27
*Lacticaseibacillus paracesei*	0	0	0	0	0	0	0	0	0	0	0	0	0	0	0
*Escherichia coli*	10	10	10	10	10	10	10	10	10	10	10	10	10	10	10
*Salmonella* Enteritidis	10	10	10	10	10	10	12	11	12	13	15	14	11	15	14
*Pseudomonas aeruginosa*	13	14	14	15	14	10	14	13	13	15	14	12	13	12	14
*Klebsiella pneumonia*	13	13	13	14.5	13	10	13	13	12	13	14	13	12	11	15
*Saccharomyces cerevisiae*	10	10	10	10	10	10	10	10	10	10	11	11	11	11	16
*Candida albicans*	0	0	0	18	15	0	10	10	12	14	12	11	12	15	15

Inhibition zone: 5–10 mm—poor activity; 11–14 mm—moderate activity; >14 mm—strong activity.

**Table 3:** The locations of apiaries from which propolis was collected and the yields of extraction process.

Symbol of Propolis Samples	City of Apiary Localization	Provinces	Extraction Yield (%)
P1	Trójca	Lower Silesia	71.13
P2	Dąbrowa Dolna	Lower Silesia	76.81
P3	Stary Majdan	Lublin	64.20
P4	Kutno	Lódźkie	71.06
P5	Trzebinia	Lesser Poland	53.91
P6	Cegłów	Masovia	77.25
P7	Kozłowiec	Masovia	44.03
P8	Sanok	Subcarpathia	73.63
P9	Lewki	Podlaskie	51.19
P10	Sierakowo	Pomerania	60.98
P11	Kąpino	Pomerania	77.47
P12	Sosnówka	Świętokrzyskie	86.63
P13	Iława	Warmia-Masuria	68.76
P14	Kąpiel	Greater Poland	54.32
P15	Tychowo	West Pomerania	44.45

## Data Availability

Not applicable.

## Supplementary Materials

The following supporting information can be downloaded at: https://www.mdpi.com/article/10.3390/molecules28010141/s1, Figure S1: Chromatogram of standards mixture (each compound at the level—10 mg/L); 1—catechin, 2—vanillic acid, 3—syringic acid, 4—epicatechin, 5—caffeic acid, 6—sinapic acid, 7—coumaric acid, 8—3-hydroxycinnamic acid, 9—ferulic acid, 10—cinnamic acid, 11—myricetin, 12—pinobanksin, 13—naringenin, 14—quercetin, 15—CAPE, 16—pinocembrin, 17—apigenin, 18—kaempferol, 19—pinostrobin, 20—chrysin, 21—galangin; Table S1: Identification parameters of phenolic compounds (retenion time, m/z, linear range, r^2^, limit of detection (LOD) and limit of quantification (LOQ) for UPLC/PDA/TQD method;

## Sample Availability

Samples of the propolis are available from the authors.
