# Supplementary material for: Biological Activity and Chemical Composition of Propolis from Various Regions of Poland
Source: Molecules. 2022 Dec 24;28(1):141. doi: 10.3390/molecules28010141 (PMC9822435; doi:10.3390/molecules28010141)
Supplement: Supplementary file 1 [file molecules-28-00141-s001.zip › molecules-2080499-supplementary.pdf]

Table S1. Identification parameters of phenolic compounds (retenion time, m/z, linear range, r^2, limit of detection (LOD) and limit of quantification (LOQ) for UPLC/PDA/TQD method.

Compounds	Retenion time (min)	MS[1] (m/z)	Linear range (mg/L)	R^2	LOD (mg/L)	LOQ (mg/L)
Catechin	2.21	291.2	10-50	0.9995	0.04	0.12
Vanillic acid	2.60	166.9	20-100	0.9992	0.35	1.05
Syringic acid	3.01	199.2	10-50	0.9996	0.04	0.12
Epicatechin	3.62	291.2	10-50	0.9997	0.04	0.12
Caffeic acid	4.68	178.7	20-100	0.9994	0.32	0.96
Sinapic acid	4.90	222.8	5-25	0.9998	0.02	0.06
Coumaric acid	5.54	163.0	5-25	0.9997	0.03	0.09
3-Hydroxycinnamic acid	6.01	162.7	5-25	0.9997	0.03	0.09
Ferulic acid	6.89	195.1	5-25	0.9996	0.03	0.09
Cinnamic acid	7.03	147.0	20-100	0.9997	0.12	0.36
Myricetin	8.47	316.7	20-100	0.9994	0.08	0.24
Pinobanksin	9.12	271.0	20-100	0.9993	0.30	0.90
Naringenin	9.80	270.8	20-100	0.9995	0.10	0.30
Quercetin	10.11	300.9	20-100	0.9996	0.11	0.33
CAPE	10.62	283.0	20-100	0.9994	0.08	0.24
Pinocembrin	12.49	257.0	20-100	0.9997	0.09	0.27
Apigenin	12.81	269.0	20-100	0.9998	0.08	0.24
Kaempferol	13.02	284.7	10-50	0.9998	0.04	0.12
Pinostrobin	13.31	271.1	10-50	0.9996	0.05	0.15
Chrysin	15.09	252.8	20-100	0.9995	0.15	0.45
Galangin	15.46	271.1	5-25	0.9998	0.025	0.075

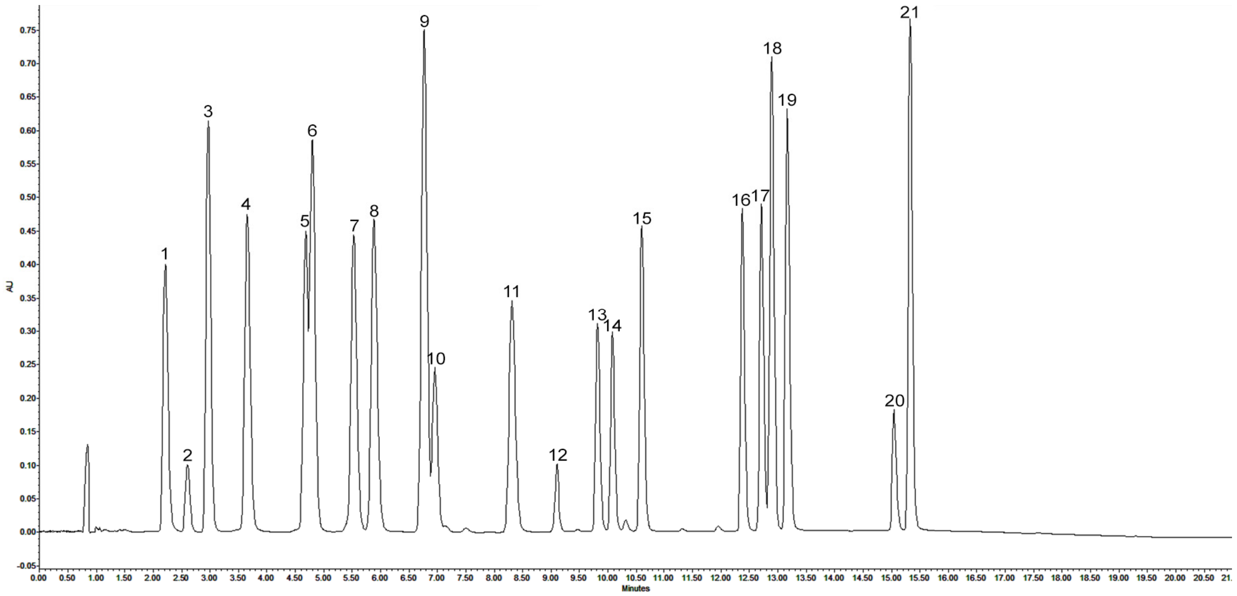

Figure S1. Chromatogram of standards mixture (each compound at the level – 10 mg/L); 1 – catechin, 2 - vanillic acid, 3 - syringic acid, 4 – epicatechin, 5 - caffeic acid, 6 - sinapic acid, 7 - coumaric acid, 8 - 3-hydroxycinnamic acid, 9 - ferulic acid, 10 - cinnamic acid, 11 – myricetin, 12 – pinobanksin, 13 – naringenin, 14 – quercetin, 15 – CAPE, 16 – pinocembrin, 17 – apigenin, 18 – kaempferol, 19 – pinostrobin, 20 – chrysin, 21 – galangin.
